# Risk factors and long-term health consequences of macrosomia: a prospective study in Jiangsu Province, China

**DOI:** 10.7555/JBR.26.20120037

**Published:** 2012-07-06

**Authors:** Shouyong Gu, Xiaofei An, Liang Fang, Xiaomin Zhang, Chunyan Zhang, Jingling Wang, Qilan Liu, Yanfang Zhang, Yongyue Wei, Zhibin Hu, Feng Chen, Hongbing Shen

**Affiliations:** a Department of Epidemiology and Biostatistics, School of Public Health, Nanjing Medical University, Nanjing, Jiangsu 210029, China;; b Department of Endocrinology, Jiangsu Province Hospital of Chinese Medicine, Nanjing, Jiangsu 210029, China;; c Department of Science and Technology, Jiangsu Population and Family Planning Committee, Nanjing, Jiangsu 210008, China;; d Genitalia Hygiene Research Center, Jiangsu Institute of Planned Parenthood Research, Nanjing, Jiangsu 210036, China.

**Keywords:** risk factors, long-term, health consequences, macrosomia

## Abstract

We sought to determine risk factors associated with fetal macrosomia and to explore the long-term consequence of infant macrosomia at the age of 7 years. A prospective population based cohort study was designed to examine the associations between maternal and perinatal characteristics and the risk of macrosomia. A nested case-control study was conducted to explore the long-term health consequence of infant macrosomia. The mean maternal age of the macrosomia group was 24.74±3.32 years, which is slightly older than that in the control group (24.35±3.14 years, *P* = 0.000). The mean maternal body mass index (BMI) at early pregnancy was 22.75±2.81 kg/m^2^, which was also higher than that in the control group (21.76±2.59 kg/m^2^, *P* = 0.000). About 64.6% of macrosomic neonates were males, compared with 51.0% in the control group (*P* = 0.000). Compared with women with normal weight (BMI: 18.5-23.9 kg/m^2^), women who were overweight (BMI: 24-27.9 kg/m^2^) or obese (BMI≥28 kg/m^2^), respectively, had a 1.69-fold (*P* = 0.000) and a 1.49-fold (*P* = 0.000) increased risks of having a neonate with macrosomia, while light weight (BMI<18.5 kg/m^2^) women had an approximately 50% reduction of the risk. Furthermore, macrosomia infant had a 1.52-fold and 1.50-fold risk, respectively, of developing overweight or obesity at the age of 7 years (*P* = 0.001 and *P* = 0.000). Older maternal age, higher maternal BMI at early pregnancy and male gender were independent risk factors of macrosomia. Macrosomic infant was associated with an increased predisposition to develop overweight or obesity at the beginning of their childhood.

## INTRODUCTION

Fetal macrosomia has attracted immense attention because of the increased risk for both mothers and infants. For mothers, it is well established that delivery of a macrosomic newborn is a risk factor for protracted labor, caesarean delivery and postpartum hemorrhage. For macrosomic infants, short-term consequence is birth trauma[1], and long-term consequences include increased predisposition to develop obesity and type 2 diabetes later in life[2]. Birth weight varies depending on several maternal characteristics, including racial origin, age, body mass index (BMI), parity and cigarette smoking. It also depends on medical conditions, such as pre-pregnancy diabetes mellitus[3]-[5].

A trend toward a higher birth weight has been demonstrated in most developed countries in recent decades[6]-[8]. Chinese national health services survey showed that birth weight increased from 3,186 g in 1993 to 3,300 g in 2008[9]. A rapid increase in the rate of macrosomia has been reported in China. For example, Bao *et al*.[10] found that the incidence of macrosomia increased from 8.31% in 2001 to 10.50% in 2005 in the city of Harbin. In Shanghai, the rate of macrosomia increased by 50% from 1989 to 1999[11]. However, few studies were performed on the contributions of risk factors to the increased incidence of macrosomia and the long-term health risks in adulthood and even childhood. In the present study, a population-based survey was therefore conducted to examine risk factors for macrosomia in Jiangsu province, China. We also explored the long-term health consequences of infant macrosomia.

## SUBJECTS AND METHODS

### Subjects

Ninety-five communities were randomly selected as surveillance spots by stratified cluster sampling in Jiangsu province, China. All pregnant women in the communities at the first trimester were investigated. Each woman was assigned a unique identification number when she was at the first prenatal care visit. The women were followed up during their pregnancy, delivery and immediate postpartum period by local family planning service professionals. We collected information on parental demographics, maternal medical, reproductive history, and medical conditions during pregnancy and pregnancy outcome (such as gestational age, birth weight, birth length, gender of baby, and congenital anomalies). In 2010, we conducted a cross-sectional study of birth cohort that consisted of macrosomia and the controls delivered in 2003. All information on the children's growth and development (weight, height) were collected. The protocol was approved by the local institutional review boards of each author's affiliated institutions, and all subjects provided signed informed consent.

Women with multiple pregnancies, preterm births, and insufficient information on birth weight at term were not included in this present study. Cases with congenital malformations and low birth weight were excluded from the study. Any normal singleton baby delivered at term that weighed 4,000 g or more was classified as macrosomic, irrespective of gestational age[12]. We carried out a comparison of factors related to macrosomia between 2,488 macrosomic newborns and a control group of 18,827 newborns, who weighed from 2,500 g to 3,999 g, using an unmatched casecontrol study design. We also performed a comparison of the development at the age of 7 years between 700 children with macrosomia and a control group of 5137 with normal birth weight from the birth cohort, by using an unmatched nested case-control study design. The study was approved by Jiangsu population and family planning committee. Written informed consent was obtained from the participants or their legal surrogates.

We examined risk factors for macrosomia in the context of maternal age, maternal education, maternal residence, maternal BMI at early pregnancy (within 12 weeks of gestation), maternal smoking/drinking during pregnancy, and infant gender. Maternal education was categorized as elementary school or less, junior middle school, high school or above. Maternal BMI at early pregnancy was based on measured height and weight at the first prenatal visit during the first trimester. According to the Group of China Obesity Task Force reference[13], maternal BMI was grouped into four categories: < 18.5 kg/m^2^, 18.5-23.9 kg/m^2^, 24-27.9 kg/m^2^, and ≥28 kg/m^2^; BMI for boys at the age of 7 years was grouped into three categories: normal (< 17.4 kg/m^2^), overweight (17.4-19.2 kg/m^2^), and obesity (≥19.2 kg/m^2^); BMI for girls at the age of 7 years was grouped into three categories: normal (< 17.2 kg/m^2^), overweight (17.2-18.9 kg/m^2^), and obesity (≥18.9 kg/m^2^).

### Statistical analysis

Continuous data were described as mean±standard deviation (SD), and categorical data were described as proportion. Continuous variables in two independent groups were compared by Student's *t* test. The chi-square test and rank sum test were used when comparing dichotomous and rank data separately. Logistic regression was used to examine the associations between maternal and perinatal characteristics and the risk of macrosomia. Odds ratio (OR) with 95% confidence interval (95% CI) for each candidate factor was calculated. *P* < 0.05 was considered as statistically significant. Statistical analyses were conducted using SAS Version 9.13 (SAS Institute Inc., Cary, NC, USA).

## RESULTS

There were 27,001 live births from December 1, 2002 to May 31, 2005 in our study sites. We excluded 5149 births with congenital malformations, or missing birth weight, or gestational age values outside the range of 20-44 weeks. After exclusion of 537 multiple births and low birth weight infants (< 2,500 g), there were 21,315 live-born singletons≥37 gestational weeks. In total, 21,315 maternal and neonatal records were analyzed. Among these newborns, 2,488 (11.67%) had macrosomia, and 417 (1.96%) had a birth weight of ≥4500 g. The mean weight of all newborns was 3,468±419 g. The mean weight of newborns in the macrosomia group and in the non-macrosomia group was 4,207±347 and 3,371±318 g, respectively.

***Table 1*** shows maternal and fetal characteristics between the macrosomia and control groups. The mean maternal age of the macrosomia group was 24.74±3.32 years, which was older than that in the control group (24.35±3.14 years, *P* = 0.000). The mean maternal BMI at early pregnancy was 22.75±2.81 kg/m^2^, which was also higher than that in the control group (21.76±2.59 kg/m^2^, *P* = 0.000). About 62.5% of the macrosomic neonates were males, compared with 51.0% in the control group (*P* = 0.000). There was no statistical difference in maternal residence, maternal education, and smoking (or drinking) during pregnancy.

By univariate logistic regression analyses, we found that maternal age at delivery, first trimester maternal BMI, and infant male gender were significantly associated with the risk of neonate macrosomia (***Table 2***). There was no statistically significant association between macrosomia risk and other factors such as maternal residence and maternal education. Multiple logistic regression analyses showed that maternal age at delivery, first trimester maternal BMI and infant gender were independent risk factors for macrosomia. Compared with women with normal weight (BMI: 18.5-23.9 kg/m^2^), women who were overweight (BMI: 24-27.9 kg/m^2^) and obese (BMI≥28 kg/m^2^), respectively, had a 1.69-fold (95%CI: 1.51-1.88) and a 1.49-fold (95%CI: 1.31-1.69) risk of delivering a neonatal macrosomia. Compared with female newborns, male newborns had a 1.61-fold (95%CI: 1.47-1.75) risk of being macrosomic.

**Table 1:** Maternal and fetal characteristics in the control group and the macrosomia group

Maternal characteristics	Macrosomia (*n* =2488)	Control (*n*=18827)	*P*
Maternal age at delivery	24.74 ± 3.32	24.35 ± 3.14	0.000**
Maternal BMI at early pregnancy (kg/m^2^)	22.75 ± 2.81	21.76 ± 2.59	0.000**
Infant gender			0.000**
Male	1,556(64.64%)	9,575(51.00%)	
Female	928(37.36%)	9,198(49.00%)	
Maternal residence			0.231**
Urban area	505(20.30%)	3,631(19.29%)	
Rural area	1,983(79.70%)	15,196(80.71%)	
Maternal education			0.768**
Elementary school or less	334(13.45%)	2,571(13.69%)	
Junior school	1,309(52.72%)	9,992(53.20%)	
High school or above	840(33.83%)	6,219(33.11%)	
Smoking during pregnancy			0.772**
No	2,473(99.40%)	18,664(99.22%)	
Yes	15(0.60%)	150(0.78%)	
Drinking during pregnancy			0.202**
No	2,426(98.14%)	18,337(98.48%)	
Yes	46(1.86%)	284(1.52%)	

*for Student's t-test;**for Chi-squared test. BMI: body mass index.

**Table 2:** Univariate logistic regression analysis for the association of macrosomia

Maternal and fetal characteristics	Macrosomia (*n* =2,488)	Control (*n* =18,827)	OR(95%CI)	*P*
Maternal age at delivery	24.74±3.32	24.35 ± 3.14	1.04(1.02-1.05)	0.000
Maternal BMI at early pregnancy (kg/m^2^)				
< 18.5	80(3.22%)	1,388(7.37%)	0.49(0.39-0.61)	0.000
18.5-23.9	1,519(61.05%)	12,830(68.15%)	Ref	-
24-27.9	540(21.70%)	2,631(13.97%)	1.73(1.56-1.93)	0.000
> 28	349(14.03%)	1,978(10.51%)	1.49(1.31-1.69)	0.000
Infant gender				
Male	1,556(64.64%)	9,575(51.00%)	Ref	-
Female	928(37.36%)	9,198(49.00%)	0.62(0.57-0.68)	0.000
Maternal residence				
Urban area	505(20.30%)	3,631(19.29%)	Ref	-
Rural area	1,983(79.70%)	15,196(80.71%)	0.94(0.85-1.04)	0.231
Maternal education				
Elementary school or less	334(13.45%)	2,571(13.69%)	Ref	-
Junior school	1,309(52.72%)	9,992(53.20%)	1.01(0.89-1.15)	0.898
High school or above	840(33.83%)	6,219(33.11%)	1.04(0.91-1.19)	0.909
Smoking during pregnancy				
No	2,473(99.40%)	18,664(99.22%)	Ref	-
Yes	15(0.60%)	150(0.78%)	0.84(0.25-2.77)	0.772
Drinking during pregnancy				
No	2,426(98.14%)	18,337(98.48%)	Ref	-
Yes	46(1.86%)	284(1.52%)	1.23(0.89-1.68)	0.202

BMI: body mass index.

In the nested case-control analysis by 2010, the mean weight for boys in the macrosomia group was 25.47±3.68 kg and in the control group was 24.63±3.87 kg. The difference between the two groups was statistically significant (*P* = 0.000). Similarly, the mean weight of the girls in the macrosomia group was heavier than that in the control group (24.43±3.61 kg versus 23.48±3.56 kg, *P* = 0.000). The mean BMI of boys and girls in the macrosomia group was higher than in the non-macrosomia group (boys, 17.31±2.43 kg/m^2^ *vs* 16.87±2.34 kg/m^2^, *P* = 0.000; girls, 16.77±2.04 kg/m^2^ *vs* 16.30±2.12 kg/m^2^, *P* = 0.000). Compared with the non-macrosomia group, macrosomic infant had a 1.52-fold (*P* = 0.001) and 1.50-fold (*P* = 0.000) risk, respectively, to developing overweight or obesity at the age of 7 years. After stratification by gender, we found that male macrosomia had a 1.53-fold and a 1.49-fold risk of developing overweight and obesity at the age of 7 years, respectively. The risk of developing overweight and obesity in female macrosomia was significantly higher than that in the female unaffected group (OR=1.45; 95%CI: 1.06-1.99).

## DISCUSSION

The present study has confirmed that the birth of macrosomic neonates was related to certain maternal and fetal characteristics in Chinese population. The results that the risk for macrosomia increases with maternal BMI at early pregnancy, maternal age and male gender are compatible with the findings of other investigators. Recent studies have suggested that high pre-pregnancy BMI was the most important predictor of delivering an infant with macrosomia[14]–[18]. The magnitude of effect of maternal BMI on the risk of macrosomia in non-diabetic pregnancies varies considerably between different studies and has been reported to range from 1.4- to 18-fold. Our result on maternal BMI at early pregnancy is consistent with these reports. We also found that mothers delivering macrosomic infants were significantly older (*P* = 0.000). This finding agrees with most domestic and foreign scholars' reports[10],[19]–[21]. However, Adesina *et al*.[22] in Ibadan, Nigeria, did not find any significant difference in maternal age. There was a male predominance (64.6%) in our study group. This was also reported by Wollschlaeger[19] from Germany and Tomic[23] from Bosnia.

**Table 3:** Long-term health consequences of macrosomia for children at the age of 7 years

	BMI	Macrosomia (*n* =700)	Control (*n* =5137)	OR (95%CI)	*P*
Male	Normal	259(60.66%)	1,838(69.99%)	-	-
	Overweight	99(23.19%)	459(17.52%)	1.53(1.19-1.97)	0.001
	Obesity	69(16.15%)	328(12.49%)	1.49(1.14-1.99)	0.007
Female	Normal	183(67.03%)	1,873(74.56%)	-	-
	Overweight	58(21.24%)	408(16.24%)	1.45(1.06-1.99)	0.019
	Obesity	32(11.73%)	231(9.20%)	1.42(0.95-2.11)	0.087
Total	Normal	442(63.14%)	3,711(72.24%)	-	-
	Overweight	157(22.43%)	867(16.88%)	1.52(1.24-1.86)	0.001
	Obesity	101(14.43%)	559(10.88%)	1.50(1.19-1.92)	< 0.001

BMI: body mass index.

Furthermore, we found that macrosomic infants had an increased predisposition to develop overweight and obesity. Compared with the non-macrosomia group, macrosomic infant had a 1.52-fold (*P* = 0.001) and 1.50-fold (*P* = 0.000) risk, respectively, of developing overweight or obesity at the age of 7 years. This is also illustrated by the data indicating that exposure to a diabetic state in utero, apparently independent of genetic factors, increases the risk of obesity and diabetes in the next generation[24]–[26]. Catalano pointed out that a vicious cycle may be established with profound consequences for the health of future generations[27].

Our study has important significance. The population is a large sample of 21,315 mother-child pairs, and the children were prospectively followed and assessed for obesity 7 years after birth. On the other hand, this study also has some limitations. Our surveillance data did not record gestational age based on ultrasound dating. Gestational age based on the first date of last menstrual period has errors, particularly among preterm and post term births[28]. In addition, pre-pregnancy BMI and weight gain during pregnancy were not recorded routinely in this study. We used maternal height and weight during the first trimester to calculate early pregnancy BMI, which was affected by both gestational weight gain and pre-pregnancy BMI. Finally, although reduction of maternal smoking during pregnancy is an important factor for macrosomia increase in developed countries[14],[17],[29],[30], we did not find statistically significant association between smoking in pregnancy and macrosomia. The prevalence of smoking in pregnancy in our population was too low (0.77%; 165 out of 21,315 women) to analyze its relationship with the occurrence of macrosomia.

In conclusion, older maternal age, higher maternal BMI at early pregnancy and male gender are independent risk factors of macrosomia. Macrosomic infants show an increased predisposition to develop overweight or obesity at the beginning of their childhood.
